# Type 2 Diabetic Patients with Ischemic Stroke: Decreased Insulin Sensitivity and Decreases in Antioxidant Enzyme Activity Are Related to Different Stroke Subtypes

**DOI:** 10.1155/2013/401609

**Published:** 2013-06-13

**Authors:** Aleksandra Jotic, Nadezda Covickovic Sternic, Vladimir S. Kostic, Katarina Lalic, Tanja Milicic, Milija Mijajlovic, Ljiljana Lukic, Milorad Civcic, Emina Colak, Marija Macesic, Jelena P. Seferovic, Sandra Aleksic, Nebojsa M. Lalic

**Affiliations:** ^1^Clinic for Endocrinology, Diabetes and Metabolic Disorders, Clinical Centre of Serbia, Faculty of Medicine, University of Belgrade, Dr Subotica 13, 11000 Belgrade, Serbia; ^2^Clinic for Neurology, Clinical Centre of Serbia, Faculty of Medicine, University of Belgrade, Dr Subotica 6, 11000 Belgrade, Serbia; ^3^Institute of Medical Biochemistry, Clinical Centre of Serbia, Pasterova 2, 11000 Belgrade, Serbia

## Abstract

We analyzed (a) insulin sensitivity (IS) and (b) glutathione peroxidase (GSH-Px), glutathione reductase (GR), and superoxide dismutase (SOD) antioxidant enzyme activity in type 2 diabetic (T2D) patients with atherothrombotic infarction (ATI) (group A), lacunar infarction (LI) (B), or without stroke (C) and in nondiabetics with ATI (D), LI (E), or without stroke (F). ATI and LI were confirmed by brain imaging IS levels were determined by minimal model (Si index), and the enzyme activity by spectrophotometry. In T2D patients, Si was lower in A and B versus C (1.14 ± 0.58, 1.00 ± 0.26 versus 3.14 ± 0.62 min^−1^/mU/l × 10^4^, *P* < 0.001) and in nondiabetics in D and E versus F (3.38 ± 0.77, 3.03 ± 0.72 versus 6.03 ± 1.69 min^−1^/mU/l × 10^4^, *P* < 0.001). Also, GSH-Px and GR activities were lower in A and B versus C (GSH-Px: 21.96 ± 3.56, 22.51 ± 1.23 versus 25.12 ± 1.67; GR: 44.37 ± 3.58, 43.50 ± 2.39 versus 48.58 ± 3.67 U/gHb; *P* < 0.001) and in D and E versus F (GSH-Px: 24.75 ± 3.02, 25.57 ± 1.92 versus 28.56 ± 3.91; GR: 48.27 ± 6.81, 49.17 ± 6.24 versus 53.67 ± 3.96 U/gHb; *P* < 0.001). Decreases in Si and GR were significantly related to both ATI and LI in T2D. Our results showed that decreased IS and impaired antioxidant enzymes activity influence ischemic stroke subtypes in T2D. The influence of insulin resistance might be exerted on the level of glutathione-dependent antioxidant enzymes.

## 1. Introduction 

It was previously suggested that atherothrombotic infarction (ATI) and lacunar infarction (LI), as two different subtypes of ischemic stroke, might also differ in the set of the relevant risk factors, with ATI being more associated to the atherogenic risk factors in contrast to LI [1]. In addition, the risk factors for both ischemic stroke subtypes remain still largely unclarified. 

However, decreased insulin sensitivity (IS), that is, insulin resistance, was observed both in ATI and LI, which was frequently accompanied with compensatory hyperinsulinemia in T2D patients as well as in nondiabetics [2, 3].

Simultaneously, it has been shown that impaired balance between products of oxidative stress and the level of antioxidant enzyme activities might be the important mechanism underlying the occurrence of ischemic stroke [4]. Moreover, it was elucidated that the brain has only moderate content of glutathione-dependent enzymes, for example, glutathione peroxidase (GSH-Px), glutathione reductase (GR), and superoxide dismutase (SOD), together with the fact that the intact antioxidant defense could provide first line of protection from initiation and exacerbation of ischemic cerebral injury [5]. 

In addition, changes in enzymatic antioxidative defense mechanisms in patients with stroke are still controversial. Previous results implied that the majority of antioxidant enzyme activity was significantly reduced in acute ischemic stroke, possibly as a consequence of increased oxidative stress [5] while the recent finding suggested increased levels of glutathione dependent enzymes as an adaptive mechanisms during acute cerebral ischemia [6]. Finally, due to novel facts, oxidative stress can be an important component for astrocytic cell death following metabolic stress [7]. 

Until now, experimental studies provided evidence of an association between ischemic stroke and increased oxidative stress [8, 9], but data in humans are still heterogeneous and limited. Therefore, our study was aimed to determine IS levels and three different types of antioxidant enzyme activities GSH-Px, GR, and SOD, in T2D with ATI and LI.

## 2. Materials and Methods

### 2.1. Patients

In this study we included a total of 93 patients with T2D, ascribed to the following groups: T2D patients with ATI (group A, *N* = 30), and T2D with LI (group B, *N* = 30), and T2D without ischemic stroke (group C, *N* = 33). Simultaneously, we involved a total of 93 nondiabetics, matched with the T2D patients regarding gender and age, and also comprising the following groups: nondiabetics with ATI (group D, *N* = 30), nondiabetics with LI (group E, *N* = 30), and nondiabetics without stroke (group F, *N* = 33). 

T2D was diagnosed in accordance with the criteria of the World Health Organization [10]. Diagnosis of ATI and LI was done by a neurologist due to clinical features and brain imaging methods such as cranial computerized scan and/or magnetic resonance imaging in two consecutive examinations, during the first 7 days from the appearance of ischemic stroke [11]. The patients with ATI or LI were included in the study provided that they had not shown signs of cardioembolic cerebral infarction, or coronary heart disease based on a history of myocardial infarction with definite elevation of serum cardiac enzymes or coronary angiography. T2D patients were treated with insulin therapy, and/or ingestion of antioxidant supplements and drugs, which might affect free radical and antioxidant activity potential; likewise patients who had other endocrine disease or autoimmune diseases, renal or hepatic failure, current infections, neoplasms, polycythemia, or rheumatic diseases were also excluded as well as the patients with history of trauma or operation within the last 3 months. No patient had uncontrolled hypertension, severe alcohol consumption, acute infection, or an inflammatory disease during the last 4 weeks. All the patients, with or without ATI and LI, showed the similar level of their physical activity. In addition, they were required not to smoke at least 12 hr before the tests were performed.

The patients were fully informed about the study and gave the inform consent to participate. 

The study was conducted at the Clinic for Endocrinology, Diabetes and Metabolic Diseases and at the Clinic for Neurology, Clinical Centre of Serbia, Faculty of Medicine, University of Belgrade, and was approved by the Institutional Ethics Committee.

### 2.2. Study Design

The interview, physical examination, metabolic test, and evaluation of antioxidant enzyme activities were performed in all the patients included in the study, for each patient within the same day. The interview comprised the questions about medical conditions, current medication, and habits. Hypertension was diagnosed according to World Health Organisation criteria (systolic/diastolic blood pressure ≥140/≥90 mm Hg) or by the use of antihypertensive agents [12].

### 2.3. Metabolic Evaluations

The metabolic tests were implemented at least after 6 months from the occurrence of the ischemic stroke. 

 The evaluation of insulin sensitivity was done by Frequently Sampled Intravenous Glucose Tolerance Test (FSIGT) with minimal model analysis [13]. Briefly, before testing, each patient was required to be at a 12 hr fasting state. During the FSIGT, 0.3 g/kg body weight of glucose was injected and the blood samples for plasma glucose (PG) and plasma insulin (PI) determination were taken immediately before and 1, 2, 3, 4, 5, 6, 7, 8, 9, 10, 12, 14, 16, 20, 23, 24, 25, 27, 30, 40, 50, 60, 70, 80, 90, 100, 120, 160, and 180 minutes after intravenous glucose stimulation. Insulin was injected as a continuous infusion 4 mU/kg/min between minutes 20 and 25 in order to avoid the effect of the potentially blunted insulin response. The insulin sensitivity index (Si) was calculated from the results of PG and PI levels by computerized minimal model analysis, using the MINMOD program (kindly provided by Dr. Richard Bergman from the University of Southern California, Los Angeles) [13]. 

### 2.4. Laboratory Analyses

Determination of the antioxidant enzymes SOD, GSH-Px, GR was conducted using the commercial assays (produced by Randox Laboratories Ltd., UK), based on spectrophotometer determination methods as described previously [14].

PG was determined by glucose oxidase method using a Beckman Glucose Analyser (Beckman Instruments, Fullerton, CA). PI was tested by radioimmunoassay (INEP, Zemun, RS, double antibody kits). Total cholesterol, HDL cholesterol, and triglyceride concentrations were determined with the chromatography method using commercial kits (produced by Boehringer Mannheim). LDL cholesterol concentrations were calculated using the Friedewald formula.

### 2.5. Statistical Analyses

Data are presented as mean ± SE. The categorical variables were analyzed with Kruskal-Wallis Test. The continuous variables within each subtype of ischemic stroke were analyzed with analysis of variance (ANOVA) with a post hoc Bonferroni test. Multiple logistical regression analysis was performed. Differences were considered statistically significant at *P* < 0.05. All analyses were performed with the SPSS statistical package (version 16.0 for Windows).

## 3. Results

### 3.1. Clinical Characteristics

The clinical characteristics and biochemistry parameters of T2D patients and nondiabetics with ATI or LI as different subtypes of ischemic stroke are shown in Table 1. The age, gender, duration of diabetes, and duration from the onset of ischemic stroke were similar in T2D patients and nondiabetics with different subtypes of ischemic stroke, together with HbA_1_c levels, implying satisfactory metabolic control before metabolic investigation was done. However, LDL-c level was significantly higher in T2D patients with ATI compared to the T2D patients with LI and T2D patients without stroke and also in nondiabetics with ATI compared to nondiabetics with LI and healthy controls. There is no difference in prevalence of hypertension in patients with T2D and ATI or LI and T2D without ischemic stroke, while it was significantly higher in nondiabetics with ATI or LI than in healthy controls. The percentage of patients who were smokers was similar in all investigated groups.

### 3.2. Insulin Sensitivity

**Figure 1:**
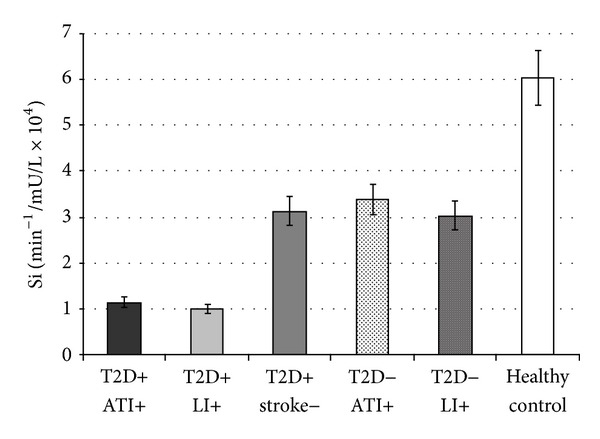
Values are expressed as mean ± SE. Bar graphs show the values of insulin sensitivity index (Si) determined by minimal model analysis. Si levels were significantly lower in type 2 diabetic (T2D) patients with different subtypes of ischemic stroke: atherothrombotic infarction (ATI) and lacunar infarction (LI) compared to T2D patients without ischemic stroke, and the same relationship is found in the respective groups in nondiabetics (*P* < 0.001) (ANOVA test).

**Figure 2:**
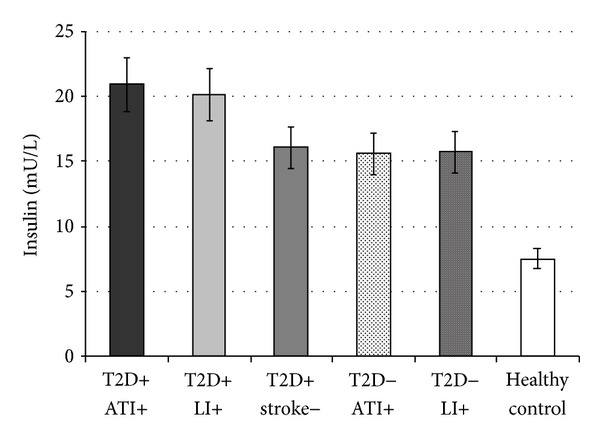
Values are expressed as mean ± SE. Bar graphs show the values of basal plasma insulin (PI) level. PI levels were higher in type 2 diabetic (T2D) patients with different subtypes of ischemic stroke: atherothrombotic infarction (ATI) and lacunar infarction (LI) compared to T2D patients without ischemic stroke, and the same relationship is found in the respective groups in nondiabetics (*P* < 0.001) (ANOVA).

We found that Si levels were significantly lower in T2D patients with ATI (group A) and LI (group B) compared to T2D patients without ischemic stroke (group C) (1.14 ± 0.58 and 1.00 ± 0.26 versus 3.14 ± 0.62 min^−1^/mU/L × 10^4^, resp., *P* < 0.001). Also, the results showed significantly lower Si levels in nondiabetics with ATI (group D) and LI (group E) compared to healthy controls (group F) (3.38 ± 0.77 and 3.03 ± 0.72 versus 6.03 ± 1.69 min^−1^/mU/L × 10^4^, resp., *P* < 0.001) (Figure 1). Simultaneously, PI levels were higher in T2D patients, ATI (group A), and LI (group B) than in T2D patients without ischemic stroke (group C) (20.94 ± 4.31 and 20.07 ± 0.88 versus 16.06 ± 0.91 mU/L, respectively, *P* < 0.001) and in nondiabetics with ATI (group D) or LI (group E) in comparison to healthy controls (group F) (15.57 ± 1.86 and 15.59 ± 1.26 versus 7.54 ± 2.03 mU/L, respectively, *P* < 0.001) (Figure 2). 

### 3.3. Antioxidant Enzyme Activity

**Figure 3:**
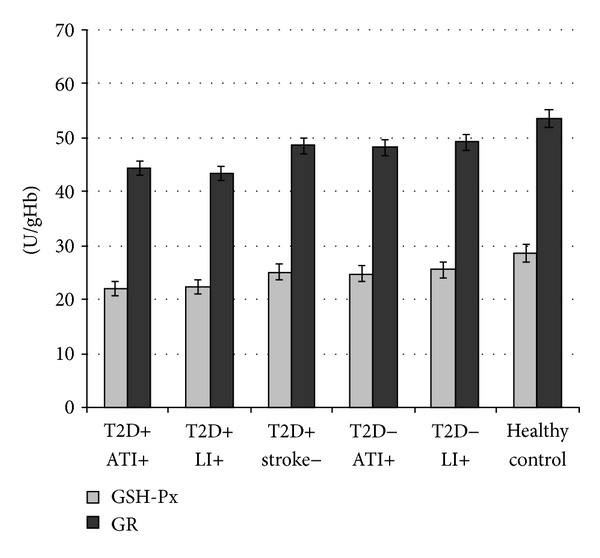
Values are expressed as mean ± SE. Bar graphs show the values of antioxidant enzyme glutathione peroxidase (GSH-Px) and glutathione reductase (GR) activity. GSH-Px and GR levels were lower in type 2 diabetes (T2D) patients with different subtypes of ischemic stroke: atherothrombotic infarction (ATI) and lacunar infarction (LI) compared to T2D patients without ischemic stroke, and the same relationship is found in the respective groups in nondiabetics (*P* < 0.001) (ANOVA test).

When we evaluated antioxidant enzyme activities in T2D patients with ATI (group A) and LI (group B) and without ischemic stroke (group C) and in nondiabetics with ATI (group D) and LI (group E) and healthy controls (group D), we detected the levels of the GSH-Px and GR activity being significantly lower in group A and B versus C and in group D and E versus F (GSHPx: A: 21.96 ± 3.56 versus B: 22.51 ± 1.23 versus C: 25.12 ± 1.67 U/gHb,  *P* < 0.001; D: 24.75 ± 3.02 versus E: 25.57 ± 1.92 versus F: 28.56 ± 3.91 U/gHb,  *P* < 0.001; GR: A: 44.37 ± 3.58 versus B: 43.50 ± 2.39 versus C: 48.58 ± 3.67 U/gHb, *P* < 0.001; D: 24.75 ± 3.02 versus E: 25.57 ± 1.92 versus F: 28.56 ± 3.91 U/gHb, resp., *P* < 0.001) (Figure 3), while the SOD levels did not differ between the investigated groups (A: 769.57 ± 72.36 versus B: 768.97 ± 34.50 versus C: 789.18 ± 60.28, D: 795.23 ± 48.28 versus E: 797.80 ± 69.21 versus F: 813.88 ± 45.80 mU/mgHb, resp., *P* = NS). 

### 3.4. Multiple Logistic Regression Analysis

This analysis identified that decreased insulin sensitivity Si and the decreases of GR were related to both ATI and LI in T2D patients. Simultaneously, this model identified decreased insulin sensitivity Si and the level of GR and GSH-Px in nondiabetics with ATI, but predominantly decreased insulin sensitivity Si in nondiabetics with LI (Table 2). 

## 4. Discussion

In this study, we directly measured the IS level, together with three different types of antioxidant enzyme activities, GSH-Px, SOD, and GR, in T2D patients and nondiabetic individuals with two different subtypes of ischemic stroke in type 2 diabetics.

Our results have shown decreased IS level in T2D patients with two different subtypes of ischemic stroke, ATI and LI, compared to T2D without stroke, while we could not demonstrate the difference between the subtypes, and the same pattern of IS changes was found in the nondiabetics. 

To our knowledge, there are scarce data regarding the changes in IS in T2D with ischemic stroke subtypes.

Previous study also suggested that insulin resistance measured using different methodology, the short insulin tolerance test, is independently associated with markers of atherosclerosis detected on carotid arteries in T2D patients [15]. Simultaneously, an association has been documented between insulin resistance and other markers at the large vessels, such as the pulsatility index on cerebral arteries [16]. On the other hand, it has been shown that insulin resistance, evaluated by the homeostasis model assessment (HOMA) index, was higher in diabetic in contrast to nondiabetic patients with LI being a small vessel disease [17]. Additionally, it has been suggested that diabetes, hypertension, and metabolic syndrome which share insulin resistance as a common mechanism, contribute to LI occurrence [18–20]. However, detailed mechanisms of the possible link between the presence of ischemic stroke subtypes and insulin resistance remain to be clarified.

In addition, when we measured the level of insulinemia in these patients, the significantly higher level of insulinemia was detected in both groups of T2D patients, with ATI and LI. The increases in insulin levels might be primarily consequence of the simultaneous presence of insulin resistance in the relevant groups. However, the increases in insulin might contribute to the appearance of the ischemic stroke subtypes. Insulin, as a growth factor, might interfere with the beneficial effects of nitric oxide (NO) on the vasculature [21]. Moreover, insulin infusion during euglycemic insulin clamp was able to suppress endothelium-dependent vasodilation in large arteries, which is reported to be based on increased availability of endothelin-1, leading to downregulation of NAD(P)H oxidase and superoxide anion production [22], and endothelial dysfunction is proposed to play an important role in the pathogenesis of cerebral small vessel disease [23, 24]. 

Also, our results demonstrated lower values of antioxidant enzymes in T2D patients with ATI or LI than in type 2 diabetics without ischemic stroke. The patients with ATI and LI did not differ regarding the level of antioxidant enzymes. These findings could be explained by the fact that in diabetes there is already reduced capacity of antioxidant protection, which is further significantly disturbed in the acute phase of ischemic stroke [4, 5].

In our data, in patients with ATI and LI, we found the decreases in glutathione-dependent enzymes activity in contrast to other types of antioxidant enzyme activity, for example, SOD. These results imply a prolonged and severe depression of gluthatione dependent antioxidative defense mechanisms irrespective of stroke subtypes. 

Since it has been documented that free radicals are extremely difficult to measure directly, antioxidant enzymes have been proposed to represent indirect markers of oxidative stress [5]. 

Numerous, but mostly experimental, studies provided evidence of an association between ischemic stroke and decreased antioxidant enzyme activity, although this possible association in humans has been less investigated [9, 25]. Moreover, the analyses of treatment with agents in stroke showing an ability to prevent further depression of antioxidant protection and scavenging reactive free radicals were reported to fail to restore GSH-Px and GR activities [26, 27].

Generally, a recently study that aimed to assess total antioxidant capacity and oxidative stress in diabetic and nondiabetic acute stroke patients with 2 different stroke subtypes, large and small vessel disease strokes, concluded that oxidative stress and counterbalancing antioxidant capacity are more pronounced in diabetic acute stroke patients than in nondiabetics [28].

The study provided different data in comparison to the previous investigations showing decreased GSH-Px activity in both diabetic and nondiabetic patients with coronary heart disease when compared to controls [17, 29, 30]. 

However, the results from our study have shown the diminished activity of both GSH-Px and GR in T2D patients with ATI and LI, which is consistent with previous reports of decreased GSH-Px levels patients with stroke [6, 8, 31, 32]. 

The levels of SOD are found to exhibit great variations in the previous study in patients with the stroke [6, 8, 26, 29, 31–37]. Our results could not detect the differences in the SOD levels in different groups of patients, and thus they are in line with data showing no changes in respect to SOD level in both diabetics and nondiabetics irrespective of different subtypes of ischemic stroke [33, 34, 37]. The tentative explanation for the inconsistent findings regarding SOD might reflect the predominant role of intracellular versus extracellular fraction of SOD in free radical scavenging [38].

In our study, the detected antioxidant enzyme activities were not affected by other important factors potentially influencing the enzyme levels, for example, by hyperglycemia, aging, duration of diabetes, and presence of macrovascular complications, due to the fact that in all groups of T2D patients had similar levels of metabolic and satisfactory metabolic control and that patients were matched in respect of age, duration of diabetes, and the prevalence of macrovascular complications. 

The multiple regression analysis applied to our data has demonstrated that decreased IS together with the decreases in GR are related to both ATI and LI in T2D patients. In nondiabetics, the decreases in Si levels and the diminished GR are found to be related only to ATI. The analysis reveals the potential difference in the mechanisms underlying the onset of the two subtypes of the stroke in T2D patients compared to nondiabetics. The results imply that higher levels of insulin resistance combined with lower levels of GR, detected in T2D patients compared to nondiabetics, were underlying the onset of LI together ATI in T2D in contrast to the findings in nondiabetics. In this context, our results are consistent with the findings that LI represents the most frequent ischemic stroke subtype in T2D. Taken together, our data imply that insulin resistance exerts its pathogenic influence on the level of gluthatione dependent antioxidant enzymes, especially in T2D. 

## 5. Conclusions

In conclusion, our results suggest that the presence of different subtypes of ischemic stroke is associated with insulin resistance and diminished antioxidant enzyme activity in both subtypes of ischemic stroke in T2D. The results also imply that atherogenic influence of decreased IS in the different subtypes of stroke in T2D might be exerted through a significantly reduced glutathione dependent antioxidant enzyme activity, while the mechanisms relating the effects of insulin resistance and decreased antioxidant enzyme activity remain to be clarified.

## Figures and Tables

**Table 1:** Clinical characteristics and laboratory analyses in type 2 diabetic patients and nondiabetics with different subtypes of ishemic stroke: atherotrombotic infarction (ATI) and lacunar infarction (LI).

	Groups						
	A	B	C	D	E	F	*P* value
	T2D+	T2D+	T2D+	T2D−	T2D−	Healthy	
	ATI+	LI+	Stroke−	ATI+	LI+	Controls	
*n* (M/F)	30 (16/14)	30 (16/14)	33 (15/18)	30 (15/15)	30 (15/15)	33 (15/18)	NS
Age (years)	56.9 ± 1.67	56.03 ± 2.51	56.42 ± 3.05	57.07 ± 2.88	56.00 ± 2.03	56.97 ± 2.42	NS
Duration of diabetes (years)	4.73 ± 1.53	5.34 ± 1.00	4.65 ± 2.01	—	—	—	NS
Duration from onset of ischaemic stroke (years)	1.23 ± 0.43	1.30 ± 0.24	—	1.03 ± 0.21	1.09 ± 0.21	—	NS
HbA1c (%)	7.39 ± 0.20	7.30 ± 0.14	7.31 ± 0.43	5.52 ± 0.31	5.37 ± 0.31	4.89 ± 0.26	NS
Total cholesterol (mmol/L)	6.98 ± 0.91	6.81 ± 0.68	7.03 ± 0.62	6.25 ± 0.71	6.15 ± 0.63	6.14 ± 0.74	NS
Triglyceride (mmol/L)	2.20 ± 0.37	2.26 ± 0.38	2.18 ± 0.26	1.90 ± 0.22	2.00 ± 0.29	1.87 ± 0.35	NS
LDL-c (mmol/L)	5.21 ± 0.42*	4.81 ± 0.14	4.47 ± 0.29	4.34 ± 0.43*	4.05 ± 0.55	3.71 ± 0.49	*P* < 0.05
HDL-c (mmol/L)	0.94 ± 0.17	1.01 ± 0.40	1.01 ± 0.15	0.99 ± 0.27	1.05 ± 0.16	1.11 ± 0.21	NS
Hypertension (*n*, %)	22 (73.3%)**	21 (70.0%)**	23 (69.7%)**	22 (73.3%)**	22 (66.7%)**	0 (0%)	*P* < 0.001
Smoking (*n*, %)	10 (33.3%)	11 (36.7%)	12 (36.4%)	10 (33.3%)	11 (36.7%)	12 (36.4%)	NS

Data are *n*, means ± SEM.

*P < 0.05 A versus B, C and D versus E, F.

**P < 0.001 A, B, C, D, E versus F.

**Table 2:** Independent factors related to different subtypes of ischemic stroke in diabetics and nondiabetics in multiple logistic regression analysis.

	Odds ratio (95% CI)	*P* value
Related to T2D+ ATI+		
Si	0.002 (0.000–0.017)	*P* = 0.000
GR	0.613 (0.422–0.889)	*P* = 0.01
GSH-Px	0.599 (0.332–1.113)	*P* = 0.105
Related to T2D+ LI+		
Si	0.001 (0.0006–0.08)	*P* = 0.000
GR	0.549 (0.369–0.817)	*P* = 0.003
GSH-Px	0.685 (0.362–1.297)	*P* = 0.245
Related to T2D− ATI+		
Si	0.159 (0.056–0.454)	*P* = 0.001
GSH-Px	0.606 (0.428–0.858)	*P* = 0.005
GR	0.795 (0.643–0.982)	*P* = 0.033
Related to T2D− LI+		
Si	0.071 (0.022–0.236)	*P* = 0.000
GSH-Px	0.760 (0.536–1.077)	*P* = 0.123
GR	0.840 (0.680–1.039)	*P* = 0.108

Nagelkerke *R* ^2^ = 0.844.

Cox and Snell *R* ^2^ = 0.821.
